# Review of brief cognitive tests for patients with suspected dementia

**DOI:** 10.1017/S1041610214000416

**Published:** 2014-03-31

**Authors:** Latha Velayudhan, Seung-Ho Ryu, Malgorzata Raczek, Michael Philpot, James Lindesay, Matthew Critchfield, Gill Livingston

**Affiliations:** 1 Department of Health Sciences, University of Leicester, Leicester , UK; 2 Institute of Psychiatry, Kings College London, London, UK; 3 Department of Psychiatry, Konkuk University Medical Centre, Konkuk University, Seoul, South Korea; 4 Old Age Psychiatry, Sussex Partnership NHS Foundation Trust, Worthing, UK; 5 Leicestershire Partnership NHS Trust, Leicester, UK; 6 Division of Psychiatry, Charles Bell House, University College London, London, UK

**Keywords:** Dementia, brief cognitive tests, cognitive screen, cognitive screening tests

## Abstract

**Background:**

As the population ages, it is increasingly important to use effective short cognitive tests for suspected dementia. We aimed to review systematically brief cognitive tests for suspected dementia and report on their validation in different settings, to help clinicians choose rapid and appropriate tests.

**Methods:**

Electronic search for face-to-face sensitive and specific cognitive tests for people with suspected dementia, taking ≤ 20 minutes, providing quantitative psychometric data.

**Results:**

22 tests fitted criteria. Mini-Mental State Examination (MMSE) and Hopkins Verbal Learning Test (HVLT) had good psychometric properties in primary care. In the secondary care settings, MMSE has considerable data but lacks sensitivity. 6-Item Cognitive Impairment Test (6CIT), Brief Alzheimer's Screen, HVLT, and 7 Minute Screen have good properties for detecting dementia but need further validation. Addenbrooke's Cognitive Examination (ACE) and Montreal Cognitive Assessment are effective to detect dementia with Parkinson's disease and Addenbrooke's Cognitive Examination-Revised (**ACE-R**) is useful for all dementias when shorter tests are inconclusive. Rowland Universal Dementia Assessment scale (RUDAS) is useful when literacy is low. Tests such as Test for Early Detection of Dementia, Test Your Memory, Cognitive Assessment Screening Test (CAST) and the recently developed ACE-III show promise but need validation in different settings, populations, and dementia subtypes. Validation of tests such as 6CIT, Abbreviated Mental Test is also needed for dementia screening in acute hospital settings.

**Conclusions:**

Practitioners should use tests as appropriate to the setting and individual patient. More validation of available tests is needed rather than development of new ones.

## Introduction

Cognitive impairment is a core and usually first symptom of dementia (APA, 1994). Efficient early diagnosis of those with suspected dementia requires quick, meaningful cognitive tests. The International Psychogeriatric Association survey found 20 brief cognitive instruments which respondents used in clinical practice chosen for “effectiveness,” “ease of administration,” and “familiarity” (Shulman *et al*., 2006). The Mini-Mental State Examination (MMSE) was the commonest, followed by the Clock Drawing Test (CDT).

Brief cognitive tests are part of the armoury required to help confirm suspected dementia and should be quick, easy, and acceptable with a high positive likelihood ratio (LR), so clinicians will be less likely to misidentify a patient with dementia. LRs are used for assessing a diagnostic test (Smith, 2009). The LR positive (LR+) is calculated as sensitivity/1-specificity and LR negative (LR−) = 1 − sensitivity/specificity. A likelihood ratio (LR) >1 indicates the test is associated with the disease (LR+), and < 1 indicates association with its absence (LR−).

There have been several narrative reviews of tests, for example, for primary care (Milne *et al*., 2008). A review of the diagnostic accuracy of longer (up to 45 minutes) tests could not identify a superior instrument (Appels and Scherder, 2010). A meta-analysis identified 15 brief cognitive tests which were less accurate than the MMSE in detecting dementia in community and primary care settings but had similar accuracy in specialist settings (Mitchell and Malladi, 2010a). Another meta-analysis examined diagnostic validity of 29 brief multi-domain screening methods and suggested alternatives tests with favourable rule-in and rule-out accuracy (Mitchell and Malladi, 2010b). However, it included tests only if administered in <10 minutes and with validation in studies with more than 170 patients. We know of no review that uses evidence-based criteria to categorize tests according to the confidence with which they can be used in the setting for which they were designed.

Our aim in this review was to identify brief cognitive tests for people with suspected dementia, and determine their level and quality of evidence in clinical settings and the types of dementia for which they are validated, in order to help clinicians choose a valid, reliable, rapid, and appropriate test most suitable for their setting. It was carried out using similar methods as earlier systematic reviews from our group (Cooper *et al*., 2011; Livingston *et al*., 2005).

## Methods

### Eligibility characteristics, information sources, and search strategy

We searched electronic databases Medline (1990–May 2013), Embase (1974–May 2013), PsychInfo (1990–May 2013), Web of Science (1990–2004), HMIC Health Management Information Consortium (1979 to March 2013) and the Cochrane library (1990–2010) for English language papers using key words—“dementia, brief cognitive tests, cognitive screen” and reference lists from included and review articles. Additionally, we hand-searched the *International Journal of Geriatric Psychiatry, Ageing and Mental Health, International Psychogeriatrics, and Age and Aging*.

### Selection criteria

We included instruments used for patients with any suspected dementia; performed solely face to face with the patient; taking ≤ 20 minutes with quantitative psychometric data and validation against dementia diagnosis (without excluding mild dementia) to include tests suitable for secondary care. We excluded tests with functional and behavioral items; telephonic or computerized self-tests, informant's questionnaires; detecting dementia praecox or dementia secondary to head injury; or mild cognitive impairment (MCI) without dementia; studies in people without dementia (unless people with dementia were analyzed separately); those to measure cognition in moderate to severe dementia rather than for suspected dementia, tested in learning disability population; qualitative tests; non-English language tests; translated versions. We also excluded tests which validation was only against other cognitive tests and those without a means of scoring for clinical practice, with no cut-off scores.

### Data extraction, quality assessment, and summary measures

Three authors, working in pairs (LV, SR, MR, MC) independently reviewed titles and abstracts for inclusion criteria. Whenever they disagreed, the full paper was reviewed with the senior author (GL). All included papers were then reread by LV, GL, or MP to ensure that they were validated against suitable criteria. We extracted data: (population, recruitment strategy, specification of illness, study design, purpose of the test, time taken to apply, total items, total scores, cut-off score, sensitivity, specificity, validity, reference standard, and blinding) and used a checklist for evaluating diagnostic tests (Table 1) (Whiting *et al*., 2004).

**Table 1.** Brief cognitive tests for dementia (Arranged by the level of evidence)

source	population, country	study design	cognitive test	illness	reference standard	loe (2011)
Crawford *et al*. (2012)	Primary and secondary care	Systematic review	ACE/ACE-R	Dementia	NINCDS-ADRDA, CDR	1
Grober *et al*. (2010)	Primary care, USA	Prospective	FCSRT	AD	CDR 0.5 to 1.0, DSM-IV	2
Larner (2007)	Memory clinic, UK	Case-control	DemTect	AD	DSM-IV	2
deKoning *et al*. (2005)	Stroke centres, the Netherlands	Consecutive	R-Camcog	Post-stroke dementia	DSM IV dementia, NINDS-AIREN	2
Borson *et al*. (2003)	Community, USA	Cohort	Mini-Cog	Dementia	DSM IIIR, CDR 1 or more, NINCDS criteria	2
Hogervortz *et al*. (2002)	Outpatients, UK	Case- control	HVLT	Dementia	DSM IV, NINCDS-ADRDA/NINDS-AIREN, CERAD,	2
Kuslansky *et al*. (2002)	Community, USA	Cross-sectional	MIS	AD	DSM-III-R, DSM-IV, NINCDS-ADRDA	2
Smith *et al*. (2007)	Memory clinic, UK	Cohort	MoCA	Dementia	ICD 10	2
Drachman *et al*. (1996)	Clinic, USA	Case-control	CAST	Dementia	NINCDS-ADRDA	2
Basic *et al*. (2009)	Clinics, Australia	Consecutive	RUDAS	Dementia	DSM IV	2
Mast *et al*. (2001)	Outpatients, USA	Cross-sectional	FOME	Dementia	NINCDS-ADRDA	2
Mitchell. (2009)	Primary and secondary care	Meta-analysis	MMSE	Dementia	NINCDS-ADRDA, DSM IV & IIIR, ICD	2
Clionsky and Clionsky (2010)	Specialist setting, USA	Consecutive	MOST	Dementia	DSM - IV	2
Hancock and Larner (2010)	Memory clinics, UK	Cross-sectional	TYM	Dementia	DSM-IV, NINCDS-ARDRA and other research diagnostic criteria for subtypes	2
Hseish *et al*. (2013)	Memory clinic, Australia	Case-control	ACE III	AD, FTD	Neuropsychological test, ACE-R	3
Mendiondo *et al*. (2003)	Memory clinic, USA	Case-control	BAS	AD	CDR, MMSE	3
Mahoney *et al*. (2005)	Community, UK	Longitudinal	TE4D-Cog test	AD	NINCDS ADRDA	3
Jitapunkul *et al*. (1991)	In-patients, UK	Consecutive	AMT	Dementia	DSM IIIR	3
Pinto and Peters (2009)	Primary and secondary care	Literature Review	CDT	Dementia	DSM IV, NINCDS-ADRDA/NINDS-AIREN	3
Brooke *et al*. (1999)	Outpatients, UK	Case-control	6 CIT	Dementia	Global deterioration scale, MMSE	3
Solomon *et al*. (1998)	Clinics, USA	Case-control	7MS	AD	NINCDS-ADRDA, MMSE, Blessed dementia rating scale	3
Kokmen *et al*. (1991)	Inpatient and outpatient settings, USA	Case-control	STMS	Dementia	DSM III R, Mattis dementia scale	3
Salib and McCarthy (2002)	Outpatients, UK	Case control	MAT	Dementia	NINCDS-ADRDA	4

AD = Alzheimer's disease; PD-D = dementia with Parkinson's disease, FTD = frontal temporal dementia. Please see Appendix for test abbreviations.

Three authors assessed study quality independently using CEBM guideline (LV, MR, SR). The senior author (GL) reviewed any disagreements. The level of evidence and grades of evidence were then assigned from standard guidelines according to the Oxford Evidence-based Medicine Centre http://www.cebm.net/index.aspx?o = 5653 (Howick *et al*., 2011). Levels of evidence (LE) ranged from one to five, with lower numbers indicating higher quality (Appendix S1). We report sensitivity, specificity and positive and negative likelihood ration where calculable or reported. Excluded tests with rationale are in Table S1. (see Table S1, FigS1, and Appendix S1 available as supplementary material attached to the electronic version of this paper at www.journals.cambridge.org/jid_IPG).

## Results

### Study selection

We identified 22 tests in the 2928 references which met inclusion criteria (Figure S1-prisma flow chart).

### Study characteristics and level of evidence

Table 1 presents the included papers with the sources and study quality. One study met criteria for the best LE (1) and 15 were rated 2 (Table 1). Most studies were carried out in USA (9) and Europe (9). Populations were usually specialist settings including memory clinics (18), followed by primary care (4), community (3) and hospital in-patient settings (2). Table 2 summarizes the tool characteristics. Table 3 gives the settings and specific dementia subtypes for which the tests were validated.

**Table 2.** Brief cognitive tests for dementia: administration times and screening performance (in alphabetical order)

Name of the test	time taken (min)	total subtests or items	total score	cut-off score (<)	sn(%)	sp (%)	+LR	-LR	IRR	TRR	suitable for visual impaired
ACE	15–20	6	100	88	93	71	3.21	0.10	ɑ	ɑ	No
ACE-R	16	5	100	82	84	100	∞	0.16	ɑ	ɑ	No
				88	94	89	8.55	0.07			
ACE-III	15	5	100	82	93	100	∞	0.07	ɑ	ɑ	No
				88	100	96	25	0			
AMT	< 10	10	10	8	91	75	3.64	0.36	ɑ	ɑ	Yes
BAS	3	4	39	26	96	98	48.0	0.02	ɑ	ɑ	Yes
CDT	1–3	1	10	7	76	78	4.00	0.3	0.88–0.97	0.94	No
CAST	15	40	43	36	88	100	∞	0.12	ɑ	0.782	No
6CIT	5	6	24	8	79	100	∞	0.21	ɑ	ɑ	Yes
Dem Tect	8–10	5	18	8	85	72	2.99	0.22	0.993	ɑ	No
FCSRT	12–15	3	48	25 (free recall)	78	90	7.80	0.24	ɑ	ɑ	No
			48	47 (cued recall)	54	90	5.40	0.6			
FOME	15	1	50	19	91	97	30.3	0.11	ɑ	ɑ	Yes
HVLT	<10	12	36	18/19	96	80	4.8	0.05	>0.99	ɑ	Yes
MoCA	10	30	30	26	94	50	1.88	0.12	0.90	0.91	No
				21	81	95	16.20	0.20			
MMSE	12	11	30	24	87	82	4.83	0.16	0.97	0.887	No
MOST	5	4	29	18	85	76	3.54	0.20	0.9	0.91	No
7MS	6–11	4		0	92	96	23.0	0.09	0.93	0.91	No
MIS	4	4	8	4	80	96	20.0	0.14	ɑ	ɑ	No
Mini-cog	2–4	2	8	5	76	89	6.91	0.27	0.93–0.95	ɑ	No
MAT	1	52	52	15	95	81	5.0	0.06	0.85	0.80	Yes
R-CAMCOG	10	25	49	34	66	92	8,25	0.37	ɑ	ɑ	No
				37	83	78	3.77	0.22			
RUDAS	10	6	30	23	88	90	8.77	0.13	0.99	0.98	No
STMS	5	8	38	31~27 (age-related)	86	92	13.29	0.15	ɑ	ɑ	No
TE4D-Cog	4–6	8	45	35	100	84	6.25	0	0.87	ɑ	No
TYM	10	10	50	42	99	86	7.07	0.01	0.99	ɑ	No

Sn = sensitivity; Sp = specificity; +LR = likelihood ratio positive; −LR = Likelihood ratio negative; IRR = Inter-Rater Reliability; TRR = Test–Retest Reliability; ∞ = insufficient or no published data available.

**Table 3.** Brief cognitive tests validated settings and specific dementia condition

i. Settings where validation tested
Primary care—GP practices
Mini-Cog, MIS, HVLT, CDT, MMSE, FCSRT
Specialist services: Memory clinics, community psychiatry services, neurology
MAT, MoCA, 6 CIT, CAST, HVLT, ACE/ACE-R, ACE-III, BAS, R-Camcog, Dem Tect, FOME, TYM, MOST, RUDAS, TED4-Cog, 7MS, STMS
Acute hospital care services
AMT, R-Camcog
ii. Tests validated for specific dementia condition
Alzheimer's disease—BAS, TE4D-Cog, Dem Tect, MIS, HVLT, FCSRT, TYM, 7MS, ACE III
Frontal lobe dementia—ACE III
Vascular—HVLT
Parkinson Disease Dementia—ACE, MoCA
Post stoke dementia—R-Camcog

### Results of individual studies

Brief descriptions of all tests follow (in alphabetical order for each section):

#### Tests validated in both primary care and specialist services

##### Addenbrooke's Cognitive Examination (ACE)

ACE is a brief test sensitive to early dementia, and differentiates dementia subtypes, including AD, FTD, Parkinson's disease dementia (PDD) and progressive supranuclear palsy (PSP) (Mathuranath *et al*., 2000; Reyes *et al*., 2009). The ACE includes the MMSE but also frontal-executive and more visuospatial items. The administration time is 16–20 minutes. The naming component has ceiling effects and the visuospatial component is relatively limited.

A cut-off at 83 gave a sensitivity of 92% and specificity of 90% in PDD, making the ACE an appropriate instrument for the first-line global evaluation of cognitive deficits in PD patients. Further studies need to evaluate the ability of the ACE to distinguish PDD from AD.

##### Addenbrooke's Cognitive Examination Revised (ACE-R)

ACE-R was derived from ACE to facilitate cross-cultural usage and improve sensitivity. The original 26 components were combined to produce five sub-scores, each representing a specific cognitive domain: attention/orientation (18 points), memory (26 points), fluency (14 points), language (26 points), and visuospatial function (16 points)–100 in total. It gives a cut-off score for the five sub-domains against controls and takes between 12 and 20 minutes (average 16). The ACE-R sensitivity to mild dementia (84% to 94% depending on cut point) is better than the MMSE (Mioshi *et al*., 2006). Three different alternative versions—A, B, and C, with different stimuli for the name and address recall, prevent recalling from previous tests. A recent systematic search of ACE and ACE-R, covering the period 2000 to April 2010, identified nine studies but none of the studies included in this review assessed inter-rater or intra-rater reliability (Crawford *et al*., 2012). The authors also highlight that there is lack of evidence on how those with vascular dementia and Lewy Body dementia perform on the ACE/ACE-R. A recent meta-analysis which reviewed the diagnostic accuracy of ACE and ACE-R reports that the ACE-R has somewhat superior diagnostic accuracy to the MMSE while the ACE appears to have inferior accuracy and that the ACE-R is recommended in both modest (primary care and general hospital settings) and high prevalence settings (memory clinics) (Larner and Mitchell, 2014).

##### Addenbrooke's Cognitive Examination (ACE-III)

In light of weaknesses of certain domains in ACE-R, such as repetition, comprehension, visuospatial, items on the ACE-R were replaced to form the ACE-III. The ACE-III continues to have a maximum score of 100 and contain five cognitive domains, but it is no longer possible to derive the MMSE score. It was tested in 61 patients with dementia (frontotemporal dementia, FTD, *n* = 33, and Alzheimer's disease, AD, *n* = 28) and 25 controls. ACE-III cognitive domains was found to correlate significantly with standardized neuropsychological tests used in the assessment of attention, language, verbal memory and visuospatial function and also compared very favorably with its predecessor, the ACE-R, with similar levels of sensitivity and specificity (Hsieh *et al*., 2013). The two tests correlated significantly (*r* p = 0.99, p < 0.01). The ACE-III also continues to show high sensitivity and specificity at cut-offs previously recommended: (1) 88 (sensitivity = 1.0; specificity = 0.96) and (2) 82(sensitivity = 0.93; specificity = 1.0). Internal reliability of the ACE-III, measured by Cronbach's α coefficient, was 0.88. It needs some training for administration and becoming familiar with the instrument usually in terms of hours. Larger studies with healthy older adults are needed in the future for age- and education-specific normative data. Also, authors suggest that utility of the ACE-III in varying clinical settings (e.g., general neurology or memory clinics) needs to be investigated and also compared with tests such as RUDAS and MoCA (Hsieh *et al*., 2013).

##### Clock Drawing Test (CDT)

The CDT is widely used, quick and non-threatening (Shulman, 2000). Probably the simplest scoring method employs a six-point rating of drawing (Shulman, 2000). It does not differentiate between Alzheimer's disease (AD), Dementia with Lewy Body (DLB), and cognitively impaired Parkinson's disease (PD) and there is little sensitivity to change (Cahn-Weiner *et al*., 2003). Validation studies are of low quality. The sensitivity (76%) and specificity (81%) for CDT are low and variable, possibly due to different patient and control groups used (Pinto and Peters, 2009). The test–retest reliability of CDT ranges from 0.87 to 0.94 and inter-rater reliability ranges from 0.82 to 0.97 depending on the scoring methods used (Manos and Wu, 1994; Seigerschmidt *et al*., 2002). Language and education influence the performance of the CDT and its use in detecting early and mild cases of dementia is limited (Pinto and Peters, 2009). Despite the various advantages of the CDT, including its simplicity, speed of administration in a busy practice and the potential to be less offensive to patients, there are still many important aspects that require further study (Pinto and Peters, 2009). These issues include: the most appropriate scoring system to be used, the training required by the rater (naive vs. professional) and at what level the test should be performed (general practitioner vs. specialized service) (Shulman, 2000; Pinto and Peters, 2009; Price *et al*., 2011).

##### Free and cued selective reminding test (FCSRT)

In the FCSRT, patients identify pictures (e.g., grapes, vest) in response to category cues (fruit, clothing) and are asked to recall them (free recall) and takes about 10–15 minutes. The category cues are used to prompt recall of items not retrieved by free recall to generate a score termed “cued recall” (Grober *et al*., 2008). Total recall is the sum of free and cued recall. Three measures derived from the FCSRT have been proposed to detect dementia: free recall, total recall and cue efficiency (the ratio of cued recall successes to the number of cued recall attempts). FCSRT has been tested both in community volunteers and in memory disorder practices (Grober *et al*., 2008; Grober *et al*., 2010). Free recall has 76% specificity and sensitivities of 83% for AD and 74% for VaD (Grober *et al*., 2008).

##### Hopkins Verbal Learning Test (HVLT)

HVLT assesses verbal recall and recognition with three learning/free-recall trials followed by a recognition trial (Rasmusson *et al*., 1995). It has six equivalent forms, for reliable re-testing even at short intervals, requires minimal training, is well-tolerated and takes under 10 minutes. It does not have ceiling effects and is not sensitive to educational levels (Frank and Byrne, 2000). The HVLT discriminated well between people with AD and controls, and was useful in clinical and epidemiological practice. In a district geriatric psychiatry service, HVLT had better sensitivity (96%) when compared to MMSE in detecting dementia with a cut off 18/19 and with high inter-rater reliability (>0.99) (Frank and Byrne, 2000). However, in a community dwelling population when tested between people with dementia and without dementia controls (including MCIs) at a cut-off of <16 the sensitivity was 80% and specificity 84%. The sensitivity increased to 90% at <18 with lower specificity 68%. Results were similar for both AD and VaD, however, when combined with WRAT-R reading (compromised in VaD), the specificity increased to 89% at a sensitivity of 90% (Kuslansky *et al*., 2004). The cut-off score of 14.5 of the HVLT “total recall” score showed a good discrimination between cases and controls (sensitivity 87% and specificity 98%). If the sensitivity needs to be higher, that is, for research, then a higher cut-off for the “total recall” of 19.5 or “memory” score with a cut-off point of 24.5 is suggested (Hogervorst *et al*., 2002).

##### Mini-Mental State Examination (MMSE)

The MMSE is a brief measure of cognitive functioning and its change, taking ≤10 minutes by a trained interviewer (Folstein *et al*., 1975). It is employed extensively in clinical settings and studies and needs some hours training and familiarizing with the instrument. The MMSE has high test–retest reliability, internal consistency and high inter-observer reliability (Folstein *et al*., 1975). There are 11 items; with maximum score of 30 and cut-off score of 24, (accounting for age, education, and language), with sensitivity of 87% and specificity of 82% (Tombaugh and McIntyre, 1992). The MMSE is short, can be used by non-specialists and its properties have been extensively studied in different populations (Nilsson, 2007). It lacks sensitivity in early dementia, FTD and dementia with Lewy bodies (DLB). It does not examine executive functions and there are few episodic and semantic memory or visuospatial tasks. Performance is affected by age, ethnicity and limited education. Consequently, the cut-point may need adjusting. For example, in highly educated persons, a cut-off of 27 yielded a sensitivity of 69% and specificity of 78% (PPV, 0.78; NPV, 0.86) for identifying dementia (Nilsson, 2007).

A meta-analysis of 34 dementia and five MCI studies using MMSE separated its use into high and low prevalence settings (Mitchell, 2009). In memory clinics the MMSE had a pooled sensitivity of 80%, in mixed specialist hospital settings 71%, in non-clinical community settings 85%, and in primary care 78%.

##### Summary

MMSE, FCSRT, CDT, and HVLT have been validated in both primary and specialist care settings. HVLT with administration time less than 10 minutes and high LR+ and low LR− currently seem best suited for both primary and secondary care settings and has better psychometric properties than the commonly used MMSE but has not been as extensively validated and only incorporates the memory domain. ACE-R is comparatively a longer test and therefore may only be appropriate in those where the diagnosis is more doubtful.

#### Tests validated in primary care

##### Mini-Cog

Mini-Cog, combines three-item word memory and clock drawing; takes about 3 minutes to perform; and was developed in a community sample that over-represented people with dementia, low education, non-white ethnicity and non-English speakers (Borson *et al*., 2000). In a population-based retrospective study, its effectiveness was also compared with MMSE and a standardized neuropsychological battery (Borson *et al*., 2003). Mini-Cog may be used successfully by relatively untrained raters as a first-stage dementia screen and its inter-rater reliability is 0.93–0.95 (Scanlan and Borson, 2001). It has lower sensitivity than the MMSE at a cut-off point of 25 (76% vs. 79%) and similar specificity (89% vs. 88%) for dementia and therefore had little advantage although it was shorter (Borson *et al*., 2003). The Mini-Cog may not be appropriate for use with patients who are visually impaired or have difficulty holding a writing implement. There are no prospective tests of its ability to detect dementia. Also the test has no value in either monitoring disease progression or rating severity.

##### Memory impairment screen (MIS)

MIS comprises four items, takes 4 minutes; and uses free and cued-recall (Buschke *et al*., 1999). The subject is asked to read the four target (to-be-remembered) aloud from a printed page. Category cues are presented then one at a time and subject are asked to identify the target word that matched the category-cue (e.g., FRUIT—PEACH). The word sheet is then removed. After a non-sematic interference task lasting 2–3 minutes, the subject is asked to recall as many of the four target words as possible (free recall) and presented with category cues for items not recalled freely (cued recall). Sensitivity was relatively low (80%) but specificity was 96% using the optimal cut-off score of four, in 438 English-speaking community volunteers (11% with dementia). Age, education, and sex did not significantly affect performance. MIS showed superior sensitivity and specificity in comparison with a three-item recall task in a population with a similar dementia prevalence and authors suggest validation in different cultural and socioeconomic setting (Kuslansky *et al*., 2002).

##### Summary

Within primary care setting where physicians are pressured for time, HVLT and MMSE are longer with the HVLT having slightly better psychometric properties. HVLT only incorporates memory and uninformative about deficits in other domains, therefore unlikely to be useful in other dementias, such as frontal lobe dementias, FTLD, and PDD. In a short consultation period, MIS, taking about 4 minutes, with high LR+ and low LR− is useful but sensitivity of only 80% means it is not good at detecting dementia.

#### Tests validated in specialist services: memory clinics, community psychiatry, neurology, and general medicine services

##### Abbreviated Mental Test (AMT)

The Mental Test Score (MTS) and its abbreviated version are brief questionnaires to assess the degree of cognitive function, particularly memory and orientation; the MTS takes 10 minutes to administer, and the abbreviated form (AMT) takes 3 minutes, is widely used, particularly in UK primary care (Hodkinson, 1972). The AMT validity was evaluated in acute geriatric ward inpatients with normal cognition, dementia and delirium (Jitapunkul *et al*., 1991). The best cut-off was 8/10 to differentiate normal from abnormal cognition including delirium, with a sensitivity of (91%) but a low specificity of 75%. Although brief, the AMT does not effectively test frontal/executive function. Although doctors often use it without training, it is important that it is interpreted the same way by all clinicians, for example, whether questions should be replaced or scored as missing or wrong in a variety of circumstances. Patients do not have to read, write, or draw anything to complete test, and so completion of the AMT is not affected by visual impairment, which is a common problem in older people.

##### Brief Alzheimer screen (BAS)

The BAS is a brief test developed using logistic regression to derive a predictive equation from MMSE and category fluency items from assessments with 406 cognitively normal people and 342 mild AD patients (Mendiondo *et al*., 2003). It has four components: three item recall, date, spelling ‘World’ backwards and category fluency, which altogether takes less than 3 minutes and total maximum score of 39. In validation samples, a cut-off score of 26 resulted in 99% sensitivity and 87% specificity. Patients who scored between 23 and 26 need further cognitive testing. Authors add that the screening test cannot be considered diagnostic as many factors influence the results of test such as population selection. Of particular importance is the issue of education, which is known to affect performance on spelling “WORLD” backwards and may give false positive and negative rates. It needs to be evaluated across different populations and patients with dementia subtypes. BAS do not need patient to read, write, or draw anything to complete test, so can be used in visually impaired.

##### Cognitive Assessment Screening Test (CAST)

CAST is a paper and pencil self- administered test, tested in a small sample in a general medical clinic with relatively low validity, designed to be completed by older patients with at least some high school education in about 15 minutes (Drachman *et al*., 1996). CAST has 3 parts: Part A - ten simple questions with 28 responses (e.g., writing own name and address, copying a simple figure, etc.); Part B -five more demanding questions with 12 scored responses (e.g., naming the Senators in own state, etc.); and Part C - 13 questions regarding subjective decline in memory and competence. It takes minimal examiner time/training and there was no significant change in test–retest scores over a 12-month period (r = 0.782, p < 0.01) (Drachman *et al*., 1996; Swearer *et al*., 2002). However the authors conclude that the CAST, like other brief screening tests, is not diagnostic and designed to make an initial separation between elderly patients with cognitive impairment from those whose cognitive function is probably normal (Swearer *et al*., 2002).

##### 6-Item Cognitive Impairment Test (6CIT)

The 6CIT is a brief test taking less than 5 minutes (three orientation items, count backwards from 20, months of the year in reverse order, and learn an address) which correlates highly (r^2^ = 0.911) with the MMSE but was more sensitive in validation at detecting mild dementia and is used in primary care as well-being culturally unbiased (Brooke and Bullock, 1999). It can be used in visual impaired people to test their cognitive abilities (Rees *et al*., 2010). However, the quality of the validation is low. The number of items is low and therefore the training time should be short.

##### DemTect

DemTect is a short (8 to 10 minutes) test for dementia, comprising five short subtests (10-word list repetition, number transcoding, semantic word fluency task, backward digit span, delayed word list recall) and its transformed total score (maximum 18) is independent of age and education. It also has high test–retest and inter-rater reliability (Kalbe *et al*., 2004). It is well accepted by patients and requires little specific training to administer. It is sensitive (85%) but not very specific (72%) (Larner, 2007).The five subtests cover immediate and delayed verbal recall, working memory, language and number processing, and executive functioning. It has only been validated in a memory clinic population with high education level and with FDG-PET as reference (Scheurich *et al*., 2005; Larner, 2007).

##### Fuld object memory evaluation (FOME)

FOME evaluates encoding and retrieving ten unrelated items across five immediate recall and a delayed recall trial (Fuld *et al*., 1990). It is sensitive to changes and can differentiate those with dementia from community healthy controls (Fuld *et al*., 1990). It is highly sensitive in nursing homes, 93% but specificity is low at 64% (Mast *et al*., 2001). The performance of FOME is not influenced by age, educational level and visual impairment (Chung and W, 2009). It has excellent test–retest reliability and parallel-form reliability, with intraclass Correlation Coefficients ranging from 0.91 to 0.96 as tested in a Chinese population (Chung, 2009).

##### Mental Alteration Test (MAT)

The MAT is modeled on Trial Making Test and involves timed performance of sequencing and category-switching between numbers and letters (Salib and McCarthy, 2002). The maximum score is 52 points, with cut-off of fewer than 15 correct alternations in 30 seconds. The test classifying correctly 95% of dementia cases if the MMSE scoring <24 on is the gold standard. The false positive rate was 19%. It can be used in visually impaired patients or in those who have difficulty in using pen and paper and has good reproducibility; test–retest correlation (*r* = 0.80) and inter-rater reliability (*r* = 0.85, κ = 0.84) (Jones *et al*., 1993).

##### Montreal Cognitive assessment (MoCA)

MoCA is a 10-minute; 30-point cognitive test with executive functioning and attention tasks designed for those scoring 24–30 on MMSE (Smith *et al*., 2007). The suggested cut-off is 26 and it has adequate test–retest reliability (Nasreddine *et al*., 2005). It was prospectively validated in a UK memory clinic setting to determine its usefulness as a predictive tool for developing dementia (Smith *et al*., 2007). At 6-month follow-up MoCA detected mild dementia in people with MCI (MMSE score above 25 points) with 94% sensitivity and 50% specificity. MoCA has excellent sensitivity (97%) for detecting MCI and MCI/AD combined but poor specificity (35%) using cut-score of 26 or below (Luis *et al*., 2009). MoCA is also accurate in PD, with cut-offs of 21/30 for PDD (sensitivity 81%; specificity 95%; negative predictive value 92%) (Dalrymple-Alford *et al*., 2010).

##### Memory Orientation Screening Test (MOST)

MOST combines three-word recall, time orientation, list memory and CDT, taking under 5 minutes and maximum score of 29 (Clionsky and Clionsky, 2010). Developed and validated in old age psychiatry settings, MOST was more sensitive than MMSE and Mini-cog for detecting dementia (Clionsky and Clionsky, 2010). The MOST demonstrated very high test–retest reliability over a brief interval (mean = 66 days, SD = 61.4) with a Pearson *r* = 0.91 (p < 0.001) and high test–retest reliability (*r* = 0.62–0.77) over a longer interval (mean = 9.2 months, SD = 4.4 months), and inter-rater reliability was *r* = 0.9, which was not examined directly (Clionsky and Clionsky, 2010). MOST requires validation in other settings and diverse population.

##### Rotterdam-CAMCOG (R-CAMCOG)

In this instrument, CAMCOG, the cognitive part of the Cambridge Examination for Mental Disorders of the Elderly was adapted to reduce administration time to ten minutes, and to improve diagnostic accuracy (de Koning *et al*., 2005). The R-CAMCOG contains 25 items testing orientation, memory (recent, remote, and learning), perception and abstraction. It can be used without confounding by paresis or mild aphasia but has unacceptable trade-offs between specificity and sensitivity. It is unsuitable for moderate to severe aphasia, visually impaired and lacks executive items, which are important for subcortical vascular deficits (de Koning *et al*., 2005). This test requires accessories, such as a picture-book, which may limit its used in routine clinical practice. Training should be relatively easy and be completed in hours.

##### Rowland Universal Dementia Assessment scale (RUDAS)

RUDAS was designed as a multicultural cognitive assessment scale and validated in an Australian community sample, measuring memory, gnosis, praxis, visuospatial skills, judgement and language (Storey *et al*., 2004). It takes ten minutes to administer, requiring minimal training and with high inter-rater (0.99) and test–retest (0.98) reliabilities (Storey *et al*., 2004). Validation in a community dwelling persons recruited from clinics and healthcare programs showed a cut-off score of 23/30 had 88% sensitivity and 90% specificity (Basic *et al*., 2009). RUDAS is relatively unaffected by gender, education and first language. However, an education bias emerged in a Malayalam translated RUDAS in a South Indian population (Iype *et al*., 2009).

##### Seven minute screen test (7MS)

7MS consists of 4 tests; Benton temporal orientation, enhanced cued recall, clock drawing and verbal fluency tasks (Solomon and Pendlebury, 1998). It is brief and unbiased by education or age. It takes a mean of 7 minutes 42 seconds (range 6–11 minutes) to administer by a trained interviewer. It requires minimal training. The overall test–retest reliability for the battery of tests was high (*r* = 0.91), and inter-rater reliability was high (*r* = 0.93) (Solomon and Pendlebury, 1998). It was useful in discriminating persons with AD from cognitively intact with sensitivity of 92% and specificity of 96% (Solomon *et al*., 1998). There have been number of validation studies in other languages including for other dementias (Meulen *et al*., 2004; Ijuin *et al*., 2008).

##### Short Test of Mental Status (STMS)

Short Test of Mental Status can be administered in inpatient and outpatient settings in approximately 5 minutes, and tests orientation, attention, immediate recall, arithmetic, abstraction, construction, information, and delayed (approximately 3 minutes) recall (Kokmen *et al*., 1991). The test was administered to a group of community patients with a diagnosis of dementia and age- and sex-matched controls. Using an age-adjusted approach, sensitivity of the test to identifying dementia is 86%, with a specificity of 94%. The STMS appeared to be modestly influenced by age and education, with correlations of −0.34 (p = .0001) for age and 0.41 (p = 0.0001) for education. The study authors additionally noted that a severe language disturbance would preclude the use of the STMS.

##### Test your memory test (TYM)

TYM is a 10-item test, self-administered under medical supervision, scoring from 0 to 50 (Brown *et al*., 2009). Although it is suggested it is self-completed it requires the clinician to be present and so we regard it as face to face. Inter-rater agreement for scoring is excellent and ten minutes’ training and the scoring sheet allowed a nurse, without experience of memory clinics, to score the TYM sheets as accurately as a specialist (Brown *et al*., 2009). It includes orientation, copying, retrograde and anterograde memory, calculation, phonemic verbal fluency, similarities, object naming, visuospatial, and executive function. It was specific and sensitive for the diagnosis of AD and to detect more cases of AD than MMSE in highly educated patients in a memory clinic, including those with sensory impairments such as hearing impairment and in situations where clinician time is limited (Hancock and Larner, 2011). It requires further validation in diverse education, cultural, and care setting.

##### Test for the early detection of dementia (TE4D-Cog)

Initially developed in Germany (known as TFDD) (Ihl *et al*., 2000), it was modified for use in an English-speaking population (Mahoney *et al*., 2005). This eight-item test is scored out of 45 and has seven subscales: immediate recall, semantic memory, CDT, category fluency, orientation to time and ideomotor praxis. A cut-off of 35 gives sensitivity of 100% and specificity of 84%, in differentiating early dementia from non-dementia. The TE4D-Cog is age, gender, and education independent in people with mild dementia. It also had good concurrent validity, high inter-rater reliability, good internal consistency, can detect change and requires minimal training (Mahoney *et al*., 2005). It requires further evaluation in memory clinics and non-English-speaking populations.

##### Summary

Amongst the tests validated in secondary care, BAS and TE4D-cog have good sensitivity and specificity to detect dementia, but need more extensive validation and longitudinal studies. 6CIT is rapid and has good psychometric properties (less than 5 minutes) but requires more extensive validation studies in communities with different demographic characteristics. The TYM and 7MS look promising but need much more evidence. RUDAS (longer but more sensitive) does not require literacy and may be more useful in those who are illiterate in English. R-CAMCOG is useful for some post-stroke dementia. Like ACE, MoCA are useful to detect dementia with Parkinson's disease, and for more detailed testing for those scoring relatively highly in shorter cognitive tests.

## Discussion

The review evaluates the 22 face-to-face cognitive tests for people with suspected dementia, which take ≤20 minutes and for which data on diagnostic validity are available. The upper limit of 20 minutes includes tests suitable for secondary care, including memory clinics, and no tests of such duration are suggested for primary care. These tests are only part of a diagnostic process which also includes history and examination of mental state. The papers do not specify which health professionals can use them but our clinical experience suggests they can be used by nurses, psychologists and doctors and the highest level skill in their use is more in the interpretation rather than the administration.

The MMSE is currently the most widely-used brief cognitive test, in routine clinical practice. Psychological Assessment Resources (PAR) holds the exclusive licence for this instrument, to publish, distribute, and manage all intellectual property rights (Martin and O'Neill, 2009). This copyright is now being enforced, at $1.23 per test (Newman and Feldman, 2011). It is particularly timely to explore the alternatives, to see if it can be replaced in routine practice (Newman and Feldman, 2011).

The Hopkins Verbal Learning Test (HVLT) has been validated both in primary care and specialist care settings, especially for AD. It takes less than ten minutes and has high LR+ and low LR−. It has better psychometric properties than the MMSE. It is currently being validated in developing countries, for example, East Asia (Hogervorst, 2011). The HVLT-revised is also copyrighted by NPAR but not the HVLT. In primary care, where time is very limited for the individual patient, the 6-CIT has potential. It takes less than five minutes (three orientation items, count backwards from 20, months of the year in reverse order, and learn an address). It correlates highly with the MMSE but was more sensitive in detecting mild dementia in primary care as well-being culturally unbiased but the quality of the validation is low. The TE4D-cog has the highest sensitivity with a reasonable specificity of 84%, and TYM, 7MS, CAST, and BAS also have good psychometric properties. All require further validation.

Among the validation studies, the review on ACE/ACE-R cognitive test yielded the highest level of evidence, which concluded the ACE-R is a robust tool for discriminating between dementia and non-dementia in clinic settings (Crawford *et al*., 2012). The newly developed version ACE-III does not have the MMSE embedded. A recent report shows that it is valid cognitive test for detecting dementia syndromes—AD and FTLD (Hsieh *et al*., 2013). ACE-R has better diagnostic accuracy than ACE and MMSE, and is recommended in both modest (primary care and general hospital settings) and high prevalence settings (memory clinics) (Larner and Mitchell, 2014).

In acute hospital care there are no instruments which have high sensitivity and specificity and further work is required. 6CIT has been tested for cognitive impairment in general hospital setting, but needs validation for dementia screening (Tuijl *et al*., 2012). A recent study which compared AMT4 with AMT10 and 6CIT cognitive tests opined that additions of tests of short-term memory such as 6CIT with AMT4 are needed to enhance accuracy for detection of cognitive impairment (Locke *et al*., 2013). The Cognitive Performance Score (CPS2), which combines data from 5 items from the interRAI Acute Care (interRAI AC) (Gray *et al*., 2008), an instrument which assesses 12 domains including cognition, physical and psychosocial functioning, appears to be another useful screening tool for assessing for dementia in acutely unwell older hospitalized patients (Travers *et al*., 2013). However, the CPS2 has not been validated as a stand-alone instrument—while the CPS2 takes less than 5 minutes to be administered, it has to be administered as part of the full interRAI AC assessment which usually takes between 40 and 60 minutes depending on the complexity of the case.

To our knowledge, this is the first review of brief cognitive tests with a broad remit and which has categorized tests according to the confidence with which we can use them in the setting for which they were designed. This review focuses on patients with mild stage of dementia minimizing variation in patient population and on cognitive tests with the best available level of evidence. This allows for reasonable comparison of the diagnostic accuracy of the instruments.

### 

#### 

##### Limitations

We have tried in to account for the bias in patient samples and specify when further or different validation is needed but lack of evidence of validity in different clinical settings is not evidence of lack of validity. Also there may be good evidence of validation but the test may not be that effective, such as MIS (Kuslansky *et al*., 2002). We have focussed on dementia. For the sake of clarity and not tried to review papers for mild cognitive impairment and therefore our findings cannot be generalized to it—We have commented only on those studies which were English language of face to face tests for those with suspected dementia and with validation data. This means we have excluded those, where there were no validation studies, for example, Epidemiological Dementia Index; those for assessing moderate to severe AD, for example, Severe cognitive impairment rating scale; taking longer, for example, Alzheimer Disease Assessment Scale-Cognitive; requiring an informant questionnaire, for example, The General Practitioner assessment of Cognition; those with no cut-off scores and specificity and sensitivity, for example, Brief Kingston Standardized Cognitive Assessment—revised, Biber Cognitive Estimation Test; those validated in non-English languages, for example, Short and sweet screening instrument, time and change test, Short Cognitive Battery; and those carried out via telephone, for example, Telephone Interview for Cognitive Status (Table S1).This does not imply that these tests are not valid. The review excluded translated versions of the tests and a future review of those would be desirable.

We have estimated training time from our clinical experience but it is usually not specified and when the authors comment it needs minimal training it is unclear in which group. We think that most tests require very short training times measured in hours rather than days for clinicians without experience in this field and less for experienced clinicians.

Validation papers do not treat psychometric tests like drugs and do not report “side-effects” of the use of instruments and longer tests may distress people more, particularly if they do badly in them, as well as being impractical in resource terms. As always it is essential that clinicians are sensitive and do not persist in these circumstances unless there is unequivocal benefit.

There are few studies comparing multiple instruments. It is important to consider that results of cognitive tests can be influenced by various factors such as selection population with performance inflated by high rates of dementia in the study sample, by high average severity of cognitive impairment among affected persons, or spectrum bias, that is, the participants are often not consecutive patients, for example, those without dementia are a convenience sample (Mahoney *et al*., 2005). The reference tests in many of these studies exhibit incorporation bias, where the index and reference tests are not independent such as CDT, Mini-Cog, BAS, MOST, 7MS, TE4D-Cog (Borson *et al*., 2003; Mendiondo *et al*., 2003; Ijuin *et al*., 2008; Pinto and Peters, 2009; Clionsky and Clionsky, 2010). The reference test standard is often concurrent clinical diagnosis which although using standard criteria is not neuropathologically validated.

It is very difficult to apply levels of evidence to markedly difficult study designs (varying from case control to systematic reviews) and while we have used standardized independent criteria in a transparent fashion to produce judgments about the comparative evidence for papers this is not definitive. Some of the criteria are arguable, for example, a systematic review ranks above an individual study but it can be the case that a beautifully carried out research project, has more accurate information than a systematic review which incorporates less good papers. Nonetheless our study organizes and adds to the information available and is transparent enough to allow readers to draw their own conclusions. Each of these diagnostic tests would require diagnostic test accuracy review in its own right and available for few tests, such as ACE/ACE-R, MMSE, and CDT (Mitchell, 2009; Pinto and Peters, 2009; Larner and Mitchell, 2014).

## Conclusions

While many brief dementia tests are available, few are widely used, and many have limited evidence regarding their performance. Despite its limitations, MMSE is still the most commonly used, and is also used as a reference standard within most validation studies. Now that there are to be significant costs associated with its use, it is important to examine whether it is the best instrument available. We have highlighted tests with better psychometric properties. Practitioners need to use tests as appropriate to the setting and individual patient, since the resources (e.g., time and personnel) and goals for use of the cognitive test differs. A stepped approach may be appropriate with the use in specialist settings of a short instrument followed by a longer one. There is need for further robust validation of available tests in varied populations for different dementia syndromes, rather than development of new ones (Milne *et al*., 2008).

## Conflict of interest

GL was one of the authors of the TE4D-cog validation paper. No other conflicts declared.

## Description of authors’ roles

LV contributed to the literature search, assessing quality of evidence, planned the overall structure of the review, took the lead in writing the manuscript and producing the tables and figures into the submitted manuscript. SR, MR, and MC contributed to the literature search, assessed quality of evidence and contributed to the final version of the manuscript; MP and JL critically appraised and edited the review. GL contributed to the level of evidence quality assessment, edited and contributed to the overall strategy of the review. LV had full access to all of the data in the study and takes responsibility for the integrity of the data and the accuracy of the data analysis.

## Appendix

**Appendix.** Abbreviations of the tools (in alphabetical order)

abbreviations	
AD	Alzheimer's disease
ACE-R	Addenbrooke's Cognitive Examination Revised
ADAS-Cog	Alzheimer's Disease Assessment Scale-cognitive subscale
AMTS	Abbreviated Mental Test score
BAS	Brief Alzheimer Screen
BDS-cog	Blessed Dementia Scale
6CIT	6 Item Cognitive Impairment Test
CAST	Cognitive Assessment Screening Test
CDT	Clock Drawing Test
CDR	Clinical Dementia Rating
CERAD	Consortium to Establish a Registry for Alzheimer's Disease
Dem Tect	Dementia Detection
DSM III-R	Diagnostic and Statistical Manual of Mental Disorders, third edition, revised
DSM IV	Diagnostic and Statistical Manual of Mental Disorders, fourth edition
FCSRT	Free and cued selective reminding test
FOME	Fuld object memory evaluation
HVLT	Hopkins Verbal Learning Test
ICD 10	International Classification of Diseases, 10th revision
MAT	Memory alteration test
MIS	Memory impairment screen
MMSE	Mini-Mental State Examination
MoCA	Montreal cognitive assessment
MOST	Memory Orientation Screening Test
MDS	Movement Disorders Task Force criteria
7MS	7 minute screen
NINCDS-ADRDA	National Institute of Neurological and Communicative Disorders and Stroke and the Alzheimer's Disease and Related Disorders Association
NINDS-AIREN	National Institute of Neurological Disorders and Stroke (NINDS) and the Association Internationale pour la Recherche et l'Enseignement en Neurosciences (AIREN)
PD-D	Dementia with Parkinson's Disease
R-CAMCOG	Rotterdam- the cognitive and self-contained part of the Cambridge Examination for Mental Disorders of the Elderly
RUDAS	Rowland Universal Dementia Assessment scale
TE4D-Cog	Test for the early detection of dementia
TYM	Test Your Memory
